# Effects of Intranasal Orexin-A (Hypocretin-1) Administration on Neuronal Activation, Neurochemistry, and Attention in Aged Rats

**DOI:** 10.3389/fnagi.2019.00362

**Published:** 2020-01-22

**Authors:** Coleman B. Calva, Habiba Fayyaz, Jim R. Fadel

**Affiliations:** Department of Pharmacology, Physiology and Neuroscience, University of South Carolina School of Medicine, Columbia, SC, United States

**Keywords:** acetylcholine, aging, attention, hypocretin, intranasal, orexin

## Abstract

Cognitive function represents a key determinative factor for independent functioning among the elderly, especially among those with age-related cognitive disorders. However; existing pharmacotherapeutic tactics for treating these disorders provide only modest benefits on cognition. The hypothalamic orexin (hypocretin) system is uniquely positioned, anatomically and functionally, to integrate physiological functions that support proper cognition. The ongoing paucity of orexin receptor agonists has mired the ability to study their potential as cognitive enhancers. Fortunately, intranasal administration of native orexin peptides circumvents this issue and others concerning peptide transport into the central nervous system (CNS). To investigate the ability of intranasal orexin-A (OxA) administration to improve the anatomical, neurochemical, and behavioral substrates of age-related cognitive dysfunction, these studies utilized a rodent model of aging combined with acute intranasal administration of saline or OxA. Here, intranasal OxA increases c-Fos expression in several telencephalic brain regions that mediate important cognitive functions, increases prefrontal cortical acetylcholine efflux, and alters set-shifting-mediated attentional function in rats. Ultimately, these studies provide a framework for the possible mechanisms and therapeutic potential of intranasal OxA in treating age-related cognitive dysfunction.

## Introduction

Increased attention is being paid to how physiological and homeostatic alterations, including altered sleep patterns and unexplained weight loss, may be early indicators of subsequent cognitive decline in the elderly (Buchman et al., 2005; Grundman, 2005; Johnson et al., 2006; Cova et al., 2016). The hypothalamus, the primary central node for integration of peripheral cues indicative of physiological status, contains cell populations comprising numerous neurochemical and neuropeptide signaling molecules, including orexins (hypocretins), that regulate the endocrine, autonomic, and behavioral responses that arise from homeostatic challenges (Li et al., 2014). Neurons expressing orexin peptides and the peptide precursor, prepro-orexin, are selectively located within the posterior portion of the hypothalamus where they form a band that spans parts of the dorsomedial nucleus, the lateral hypothalamus, and the perifornical area (de Lecea et al., 1998; Sakurai et al., 1998). Orexin neurons, while confined to a small anatomical area, send widespread ascending projections to cortical and limbic regions and descending projections to brainstem regions (Peyron et al., 1998). Additionally, reciprocal innervation, arising primarily from limbic structures, provides feedback to orexin neurons (Sakurai et al., 2005; Yoshida et al., 2006). Importantly, these wide-ranging projections place orexin neurons in a unique anatomical and functional position to integrate the physiological and cognitive responses that maintain proper homeostasis (Li et al., 2014). The extensive connectome of the orexin system puts it in a prime position to activate a multitude of phenotypic neurons throughout the brain (Eggermann et al., 2001; Wu et al., 2002; Arrigoni et al., 2010; Yan et al., 2012; Burlet et al., 2018). Accordingly, the studies described here center around interactions between the orexin system and acetylcholine, glutamate, and GABA neurotransmission.

A substantial volume of evidence has demonstrated clear orexin-cholinergic interactions and their potential role in modulating attentional function. Orexins infused into the laterodorsal tegmentum, much like the basal forebrain, induce arousal and increase wakefulness (Espaa et al., 2001; Thakkar et al., 2001; Xi et al., 2001), which ultimately suggests that cholinergic innervation to the basal forebrain is vital for maintaining arousal (Yamanaka et al., 2003; Ohno and Sakurai, 2008; Zhou et al., 2015). Intrabasalis administration of orexin-A (OxA) to anesthetized animals also increases somatosensory acetylcholine release and elicits EEG readings congruent to stereotypic EEG potentials observed during arousal (Dong et al., 2006). Crucially, administration of OxA into the basal forebrain not only increases cortical acetylcholine release (Fadel et al., 2005; Dong et al., 2006) but also enhances attentional capacity (Zajo et al., 2016). Additionally, the orexin-1 receptor (Ox1R) antagonist SB-334867 abolishes stimulated acetylcholine release during feeding-related arousal (Frederick-Duus et al., 2007). Orexin neurons may also enhance attention *via* interactions with other neurochemical systems. For example, direct administration of OxA into the basal forebrain increases local glutamate efflux, which, in turn, modulates activity of basal forebrain cholinergic neurons (Fadel and Frederick-Duus, 2008). Furthermore, OxA may also enhance arousal by activating putative wake-promoting GABAergic projection neurons in the basal forebrain that preferentially synapse onto GABAergic interneurons in the cortex (Freund and Meskenaite, 1992; Henny and Jones, 2008; Arrigoni et al., 2010; Anaclet et al., 2015).

An early manifestation of age-related cognitive decline is a deterioration of attentional function (Scinto et al., 1994; Perry and Hodges, 1999; Rizzo et al., 2000; Sarter and Turchi, 2002; Oken et al., 2012). Normal attentional function requires a base state of arousal, and dysfunction of these interacting behavioral states is clearly observed in narcolepsy, which is associated with a loss of orexin neurons (Thannickal et al., 2000; Nishino and Kanbayashi, 2005; Burgess and Scammell, 2012). Indeed, the sleep/wake disruptions and attentional deficits observed in narcolepsy are distinctly similar to the behavioral changes that manifest during aging and early Alzheimer’s disease (Perry and Hodges, 1999; Sarter and Turchi, 2002; Cox et al., 2019; Taillard et al., 2019). Furthermore, anatomical evidence highlights that aged subjects exhibit selective losses of orexin neurons, orexin peptides, and/or their receptors (Terao et al., 2002; Zhang et al., 2002; Porkka-Heiskanen et al., 2004; Downs et al., 2007; Sawai et al., 2010; Kessler et al., 2011). Ultimately, these observations indicate that orexins modulate cognition during aging and may be a promising therapeutic target for early intervention.

Relative to the numerous orexin receptor antagonists (Smart et al., 2001; Scammell and Winrow, 2011; Mieda and Sakurai, 2013; Steiner et al., 2013; Roecker et al., 2016; Skudlarek et al., 2017; Recourt et al., 2019), development of orexin receptor agonists has been limited (Irukayama-Tomobe et al., 2017; Turku et al., 2017), leading to greater use of native orexin peptides to examine the role of orexins in behavior and cognition. While peripheral administration of the OxA has previously been examined in animal models (John et al., 2000; Fujiki et al., 2003; Deadwyler et al., 2007; Dhuria et al., 2009), several caveats, including peripheral side effects and low bioavailability, limit the potential for this route of administration (Hallschmid and Born, 2008). Conversely, intranasal administration of neuropeptides represents a promising treatment route that may ameliorate issues related to peripheral side effects or penetration into the central nervous system (CNS) (Hanson and Frey, 2008; Lochhead and Thorne, 2012; Meredith et al., 2015; Spetter and Hallschmid, 2015). Previous behavioral evidence also supports the use of intranasal orexin administration in treating cognitive dysfunction. Intranasal OxA improves cognitive deficits associated with sleep deprivation in rhesus macaque monkeys and also enhances attention and wakefulness in human patients with narcolepsy (Deadwyler et al., 2007; Baier et al., 2011; Weinhold et al., 2014). Furthermore, evidence in rodents from our lab and others shows that intranasal OxA increases food consumption (Dhuria et al., 2016) and efflux of glutamate and acetylcholine in the prefrontal cortex (PFC) of young animals (Calva et al., 2018). While these studies demonstrate that intranasal orexin modulates certain behavioral and neurochemical correlates of cognition, its effects on cognitive aging are presently unknown. Here, intranasal OxA administration in aged rats is combined with immunohistochemistry, *in vivo* microdialysis, and attentional-set shifting to assess the effects of intranasal OxA administration on the anatomical, neurochemical, and behavioral deficits that encompass age-related cognitive decline.

## Materials and Methods

Experimental methods, materials, and procedures for immunohistochemistry and *in vivo* microdialysis were generally as described in our published works examining intranasal OxA administration in young animals (Calva and Fadel, 2018; Calva et al., 2018). Animal care and use practices, including rationale for use and number of animal subjects, were performed in accordance with protocols written under guidelines of the National Institutes of Health Guide for the Care and Use of Laboratory Animals and approved by the Institutional Animal Care and Use Committee at the University of South Carolina.

### Animals

Male Fischer 344/Brown Norway F1 hybrid rats (FBN/F1; Harlan/NIA) were used for all experiments. Young and aged animals were approximately 3–4 months (250–300 g) and 26–28 months (550–600 g), respectively, upon arrival to the animal facility. The FBN/F1 strain is commonly utilized in neurobiology of aging studies due to its reduced vulnerability to several non-neurological age-related issues (e.g., intraperitoneal tumors) observed in other strains during the later years of life (Lipman et al., 1996; Turturro et al., 1999). Additionally, previous work from our lab utilized the FBN/F1 strain for studying interactions between aging and the orexin system (Kessler et al., 2011; Stanley and Fadel, 2012; Stanley et al., 2012; Hagar et al., 2017). Animals were housed in an environmentally controlled animal facility on a 12:12 light:dark cycle with the lights coming on at 07:00 h. All experimentation was performed during the light phase, when endogenous orexin activity (as measured by c-Fos expression) is at its circadian nadir (Estabrooke et al., 2001). All animals were allowed access to food and water *ad libitum*. Several steps were taken to curtail animal distress during these experiments, including careful monitoring of the depth of anesthesia during surgery, providing postoperative analgesics (see below), and utilization of a within-subjects multisession design for the *in vivo* microdialysis studies, thus reducing the number of animals needed to achieve equivalent statistical power. The experimenter performing intranasal administration was not blinded to treatment conditions (saline vs. OxA) during experimentation; however, the experimenter was unaware of treatment conditions while conducting counts during histological imaging.

### Stereotaxic Surgery

Under ketamine (90 mg/kg) plus xylazine (2–10 mg/kg) anesthesia, animals used for *in vivo* microdialysis received a single guide cannula (BASi, West Lafayette, IN, United States) inserted into the medial PFC at AP + 2.8 mm, L ± 0.5 mm, and DV −2.8 mm relative to bregma. Guide cannula coordinates were acquired from the Paxinos and Watson (1998) rat brain atlas. The intracerebral guide cannula was anchored in place using two to three skull screws and dental cement. Guide cannula placement was counterbalanced so that left and right hemispheres were represented equally. At the conclusion of surgery, all animals were given a single dose of the analgesic buprenorphine (0.01 mg/kg, s.c.) and were monitored until complete recovery. All animals were provided at least two full recovery days prior to starting habituation in the microdialysis bowls.

### Immunohistochemistry and Immunofluorescence

Upon arrival to the animal facility, each batch of young and aged animals was assigned to receive intranasal administration of vehicle (50 μl of 0.9% saline) or OxA (50 μl of a 100-μM solution; Enzo Life Sciences, Farmingdale, NY, United States). For each batch, both treatment conditions were equally represented; however, treatment order within each batch was pseudorandomly determined before each test day. Each animal received several days of gentle handling and habituation to intranasal saline administration prior to the test day. Briefly, each animal was loosely blanketed with a small cloth and held in a supine position so that only the animal’s snout was protruding from a small opening. No restraint or anesthesia was used during intranasal habituation or treatment. On the test day, each rat was administered 50 μl of saline or OxA. Intranasal administration of the entire 50 μl volume was delivered in four 12.5-μl aliquots (a total of 25 μl in each naris) over a 2- to 3-min period.

Treatment group assignment for all immunohistochemistry experiments was pseudorandomized for each batch of animals such that treatment order (i.e., intranasal saline or intranasal orexin) was counterbalanced and equally represented for each batch. All animals were handled and habituated to intranasal saline administration prior to the treatment day. On the experimental day, animals received their designated treatment and, 2 h later [the time point at which c-Fos expression peaks (Kaczmarek, 1992)], were euthanized under deep isoflurane anesthesia and perfused with phosphate buffered saline and 4% paraformaldehyde. After overnight post-fixation, each brain was coronally sectioned at a 50-μm thickness using a vibratome. A 1:4 serial sectioning method was utilized, thus allotting 200 μm between each representative section. Sections not immediately used for immunohistochemistry were stored in 30% sucrose/30% ethylene glycol anti-freezing solution at −20°C until use. Single- and dual-label immunohistochemistry followed similar protocols, where free-floating sections were incubated with a rabbit anti-c-Fos primary antibody (1:5,000; Millipore, Billerica, MA, United States; catalog No. ABE457; RRID:AB_2631318) for 48 h at 4°C, followed by a biotinylated donkey anti-rabbit secondary antibody (1:1,000; Jackson ImmunoResearch Laboratories Inc., West Grove, PA, United States; code No. 711-065-152; RRID:AB_2340593) for 1.5 h at 23°C (RT) and a horseradish peroxidase-conjugated streptavidin tertiary antibody (1:1,600; Jackson ImmunoResearch Laboratories Inc.; code No. 016-030-084; RRID:AB_2337238) for 1 h at 23°C (RT). Staining for c-Fos was developed with 0.3% hydrogen peroxide and nickel-cobalt-enhanced diaminobenzidine (DAB) to yield blue-black immunopositive nuclei. Dual-label staining for either choline acetyltransferase (ChAT) or parvalbumin (PV) used c-Fos-stained sections that were subsequently incubated in either a goat anti-ChAT (1:3,000; Millipore, Temecula, CA, United States; catalog No. AB144; RRID:AB_90650) or a mouse anti-PV (1:4,000; Sigma, St. Louis, MO, United States; catalog No. P3088; RRID:AB_477329) primary antibody for 48 h at 4°C. Secondary and tertiary steps followed with incubations in either an unlabeled donkey anti-goat (1:200; Jackson ImmunoResearch Laboratories Inc.; code No. 705-005-003; RRID:AB_2340384) or an unlabeled donkey anti-mouse (1:200; Jackson ImmunoResearch Laboratories Inc.; code No. 715-005-150; RRID:AB_2340759) secondary antibody for 2 h at 23°C (RT), followed by incubations in either a goat peroxidase anti-peroxidase (1:500; Jackson ImmunoResearch Laboratories Inc.; code No. 123-005-024; RRID:AB_2338953) or a mouse peroxidase anti-peroxidase (1:500; Jackson ImmunoResearch Laboratories Inc.; code No. 223-005-024; RRID:AB_2339261) tertiary antibody for 1.5 h at 23°C (RT). Immunostaining for ChAT or PV was developed with 3% hydrogen peroxide and DAB to yield brown immunopositive cell bodies. Using a 0.15% gelatin solution, sections were mounted onto slides and allowed to dry overnight before dehydration, delipidation, and coverslipping with Depex mounting medium.

To perform a qualitative examination of where intranasal OxA distributed within the brain, a subset of animals (*n* = 4, aged) received intranasal administration of either saline or a modified OxA peptide labeled with a green-fluorescent fluorophore [5(6)-FAM-(Glu1)-OxA trifluoroacetate salt; BACHEM, Bubendorf, Switzerland]. Animals receiving the fluorescein-tagged OxA peptide received 50 μl of a 500-μM dose split into four 12.5-μl increments over a 2-min period and were sacrificed 30 min post-treatment. This time point was chosen based upon a previous study showing peak appearance of ^125^I–OxA in the CNS 30 min after intranasal delivery (Dhuria et al., 2009). Perfusion and brain sectioning and tissue mounting were as described above.

### Microscopy and Imaging

Single-labeled (c-Fos) cells, double-labeled (c-Fos + ChAT or PARV) cells, and AChE background staining were visualized using a Nikon E600 microscope fitted with a CoolSNAP digital camera (Roper Scientific, Trenton, NJ, United States). Fluorescence images were visualized using a Nikon E600 microscope or a Leica SP8 multiphoton confocal microscope (Leica Microsystems, Wetzlar, Germany) equipped with LAS AF 3 analysis software (Leica Microsystems, Wetzlar, Germany). Immunoperoxidase photomicrographs were captured using IP Lab Software (Scanalytics, Trenton, NJ, United States). Images were imported into Adobe Photoshop 6.0 (Adobe Systems, San Jose, CA, United States) to adjust the image size and to make minor alterations to contrast and brightness. Brain regions where photomicrographs were captured are highlighted in the results *via* modified illustrations from the Paxinos and Watson (1998) rat brain atlas.

### *In vivo* Microdialysis

In concordance with the immunohistochemistry experiments, animals used for the *in vivo* microdialysis experiments arrived in separate batches. Beginning 3 days after cannula implantation, animals were habituated in microdialysis bowls for 3–4 days and habituated with intranasal saline for 7 days. On microdialysis days, guide cannula stylets were removed and substituted with a microdialysis probe (BASi, West Lafayette, IN, United States) that extended 2 mm past the guide cannula. Probes were perfused at a 2-μl/min flow rate with artificial cerebrospinal fluid (aCSF, pH 7.4) containing 150 mM NaCl, 3 mM KCl, 1.7 mM CaCl_2_, 0.9 mM MgCl_2_, and 4.9 mM D-glucose. Neostigmine bromide (50 nM; Sigma) was added to the aCSF to increase recovery of acetylcholine in collected dialysates. Dialysate collection started after a 3-h discard period to allow stabilization of basal efflux following probe insertion. Microdialysis sessions consisted of 1 h (4 × 15-min collections) of baseline collections followed by intranasal vehicle (0.9% saline) or OxA (100 μM; Enzo Life Sciences), administered in a total volume of 50 μl in 12.5-μl increments over a 2-min period. Dialysate collection then continued for 2 h (eight 15-min collections) post-treatment. Upon collection, dialysates were stored at −80°C until analysis using high-performance liquid chromatography (HPLC). All animals underwent two separate microdialysis sessions with an off day in-between, and experiments were counterbalanced so that half of the animals received vehicle during session one whereas the other half received OxA in session one. The day following the last microdialysis session, rats were euthanized, and their brains were processed for future probe placement verification using an acetylcholinesterase (AChE) background stain. Verification of microdialysis probe placement was performed after all HPLC samples were run for each respective animal. Any probe placement visualized outside the medial PFC excluded the animal from the study.

### HPLC and Chromatogram Analysis

Each 30-μl dialysate was split prior to analysis by HPLC with electrochemical detection (HPLC-ECD), with 20 μl analyzed for ACh and 10 μl analyzed for glutamate. ACh was analyzed using an HTEC-510 HPLC-ECD (Amuza, San Diego, CA, United States). Briefly, 20 μl of each dialysate was loaded into the AC-GEL separation column (2.0 ID × 150 mm; Amuza) maintained at a constant 33°C in combination with mobile phase (pH 8.5) containing 49.4 mM potassium bicarbonate (KHCO_3_), 134.3 μM ethylenediaminetetraacetic acid disodium (EDTA-2Na), and 1.23 mM sodium 1-decanesulfonate. After analyte separation, post-column derivatization of ACh was attained through use of an AC-ENYM II enzyme reactor (1.0 ID × 4 mm; Amuza) containing AChE and choline oxidase, generating stoichiometric quantities of hydrogen peroxide, which was detected on a platinum working electrode with an applied potential of + 450 mV. The amount of ACh in each sample was measured by comparison with a three-point external standard curve with values predicted to be in range of the collected dialysates. The limit of detection for this analysis was approximately 5 fmol/injection.

Glutamate levels in brain dialysates were analyzed using a CC-32 HPLC-ECD (BASi, West Lafayette, IN, United States) with modifications. First, 10 μl of each dialysate was loaded into the GU-GEL separation column (4.6 ID × 150 mm; Amuza) in conjunction with a mobile phase (pH 7.2) containing 60 mM ammonium chloride-ammonium hydroxide, 134.3 nM EDTA-2Na, and 686 μM hexadecyltrimethylammonium bromide. After separation, post-column derivatization of glutamate was attained using an E-ENZ enzyme reactor (3.0 ID × 40 mm; Amuza) containing glutamate oxidase, generating hydrogen peroxide proportional to the amount of glutamate present. Hydrogen peroxide was detected on a 3.0-mm glassy carbon electrode (BASi) coated with a horseradish peroxidase osmium polyvinylpyridine solution (0 mV applied potential). The amount of glutamate in each dialysate was measured by comparison with a three-point standard curve using external standards expected to be in range of the collected dialysates. The limit of detection for this method was approximately 3 fmol/injection.

### Attentional Set-Shifting Task (ASST)

In rodents, the ASST takes each animal through a series of increasingly difficult tasks in which they must dig in small containers to locate a food reward. The materials and methods for the behavioral studies using the attentional set-shifting paradigm were adapted from previous studies using similar procedures (Birrell and Brown, 2000; Barense et al., 2002; Lapiz-Bluhm et al., 2008; Snyder et al., 2012). Upon arrival, young and aged FBN/F1 rats were assigned to receive intranasal administration of either vehicle (50 μl of 0.9% saline) or OxA (50 μl of a 100-μM solution; Enzo Life Sciences, Farmingdale, NY, United States). All animals were acclimatized to individual housing for at least 4 days prior to the start of any behavioral procedures.

For approximately 1 week prior to testing, each rat was maintained on a food-restricted diet of approximately 14 g per day, such that by testing day, each rat weighed approximately 80–90% of their free-feeding body weight. Each animal received several days of gentle handling and habituation to intranasal saline administration prior to the testing day. All behavioral experiments were conducted during the light portion of the cycle, between 07:00 and 19:00 h. Our testing apparatus was a custom-built black rectangular Plexiglas arena with inner dimensions of 75L × 40W × 30H (Snyder et al., 2012). The testing arena contained a black Plexiglas removable divider to separate one-third of the arena from the remaining two-thirds of the arena. This smaller portion of the arena served as the starting box for each rat and as a holding area between each trial. Each trial began after lifting the removable divider, allowing the rats to access the remainder of the arena. The larger portion of the divided arena contained an irremovable and opaque Plexiglas panel to split the arena into two sections. These two sections served as the holding area for the digging bowls. A visual representation of the testing arena is shown in Supplementary Figure S1. The digging pots used for these experiments were small plastic containers (internal rim diameter 7 cm; depth 3.5 cm). Each digging pot was distinguished by a pair of cues along two different stimulus dimensions: (1) the digging medium held in each pot and (2) an odor applied to the rim of each pot. The relevant/irrelevant dimensions and the positive/negative cue pairs for each stage of the task are shown in Supplementary Table S1. To mark each digging pot with an odor, approximately 50 μl of extract was initially applied to the inner rim of the pot. Each digging pot received only one odor, and a different pot was used for each combination. The reward, buried approximately 2 cm beneath the surface of each medium, was a “bacon softie” broken into small pieces. For each trial, a small amount of reward was ground and applied to the surface of both pots to ensure that each animal was digging correctly based upon the positive cues rather than by smelling the food reward. After the animals were food restricted for at least 5 days, the 3-day behavioral procedure was conducted as follows. On the habituation day (Day 1), each animal was required to dig for the food reward in each pot during three trials of 5 min each, with the food reward covered with an increasing amount of sawdust during each exposure. After a consistent pattern of digging was established, the animal was placed in the testing arena and habituated in the same manner as described above. On the training day (Day 2), rats were trained on a simple discrimination (SD) task to a criterion of six consecutive correct trials. Each trial during the discrimination tasks was timed for up to 10 min. If the animal did not retrieve the reward after 10 min, a non-digging response, or “error” was recorded. Any animal with six consecutive errors was subsequently excluded from further experimentation. All rats started by learning to associate the food reward with the positive odor cue (the starting media is sawdust in both digging pots). After six consecutive correct trials, the animals were trained to discriminate between two different media to achieve a reward. All animals were trained with these odors and media with the positive and negative cues randomly assigned for each animal. Importantly, the training stimuli were never used on testing days to ensure that animals were not remembering odor or media pairings during testing day. On the testing day (Day 3), each animal was tested on a series of five increasingly difficult discrimination tasks (Supplementary Table S1) before receiving its daily ration of standard rat chow. Again, the criterion used to proceed to the next stage of the experiment was six consecutive correct digging responses. All animals were tested on the first three stages of the task: SD, compound discrimination (CD), and the intradimensional shift (IDS). At this point in the task, the animal was given its predetermined treatment (i.e., intranasal saline or intranasal OxA). This time point for treatment was based upon the *in vivo* microdialysis studies where the effect of intranasal OxA on PFC acetylcholine efflux lasted approximately 30–60 min. Because the ASST experiment lasts 2–3 h for each animal, intranasal saline or OxA was given just before the reversal learning stage (REV) of the task to allow for optimal assessment of each treatment on the more challenging and PFC-dependent task components. After intranasal administration, each animal was tested on the final two stages of the task: reversal learning (REV) and the extradimensional shift (EDS). For each animal, assignment to a positive or negative cue, relevant starting dimension, and left-right positioning of the pots within the arena were determined randomly in advance.

### Statistics and Data Analysis

For all immunohistochemistry experiments, single-labeled (c-Fos) and double-labeled (c-Fos + ChAT/PV) positive cells were counted within the confines of a reticle fixed into the eyepiece of the microscope. Counts for each brain region were determined by the total number of immunopositive nuclei/cells from two representative sections at different levels of the rostrocaudal gradient. Single-label c-Fos data were expressed as the density of immunopositive nuclei counted within the reticle area (c-Fos nuclei/mm^2^). Statistical analyses of these data utilized two-tailed unpaired *t*-tests (GraphPad Prism 8; GraphPad Software for Windows, La Jolla, CA, United States). Double-labeled neurons were expressed as the percentage of the total number of ChAT/PV neurons positive for c-Fos within the reticle area (i.e., % double-labeled neurons). Dual-label immunoperoxidase data were analyzed by two-tailed unpaired *t*-tests. A significance cutoff level of *p* < 0.05 was used for all analyses.

For the *in vivo* microdialysis data, baseline neurotransmitter efflux was obtained during the first four sample collections (i.e., time points 1–4) and averaged to yield mean basal efflux. For each sample analyzed, a raw value was obtained and expressed as pmol/20 μl for ACh and μmol/10 μl for glutamate. The graphed *in vivo* microdialysis data are expressed as a percentage of the average baseline to account for individual variation in basal neurotransmitter efflux. Data were analyzed using two-way repeated-measure ANOVAs (GraphPad Prism 8; GraphPad Software, La Jolla, CA, United States) with treatment as a within-subject variable and time as a repeated measure. Significant interactions and main effects of treatment (i.e., OxA or saline) were probed with Holm-Sidak multiple comparisons tests. A significance cutoff level of *p* < 0.05 was used for all analyses.

For the attentional set-shifting experiments, trials to reach criterion (six consecutive correct trials) were recorded for each rat. Because intranasal saline or intranasal OxA was given just before the REV stage of the ASST experiment, the first three stages and the final two stages were assessed using different statistical analyses. For the first three stages of the task (i.e., SD, CD, IDS; before treatment), the treatment groups were collapsed to assess differences in task performance between young and aged animals. These results were analyzed using two-tailed unpaired *t*-tests. For the final two stages of the task (i.e., REV, EDS; after treatment), the stages were analyzed together by three-way ANOVA (stage × treatment × x age) followed by Holm-Sidak planned comparisons to probe the source of significant main effects and interactions. Data were represented as total number of trials to reach criterion (*y*-axis) for each stage (*x*-axis). A significance cutoff level of *p* < 0.05 was used for all analyses.

## Results

### Effects of Intranasal OxA Administration on c-Fos in Aged Rats

Intranasal administration of OxA significantly increased c-Fos expression in multiple brain regions of aged animals (Table 1). In the cortex, intranasal OxA increased c-Fos expression in the prelimbic cortex (PrLC; *t*_14_ = 4.276, *p* = 0.0008) and agranular insular cortex (AIC; *t*_14_ = 3.222, *p* = 0.0061). Importantly, intranasal OxA did not globally excite all brain regions as there were no significant changes in c-Fos expression within the piriform cortex, ventral orbital cortex, nucleus accumbens shell, retrosplenial cortex, and regions CA3 and CA1 of the hippocampus. Interestingly, there was a strong trend for decreased c-Fos expression in the infralimbic cortex (*t*_14_ = 1.91, *p* = 0.0769) after intranasal OxA administration. We also considered the possibility that intranasal OxA delivery may alter c-Fos expression in brain regions outside the neocortex. Indeed, intranasal OxA also significantly increased c-Fos expression in the claustrum (Cl; *t*_14_ = 3.055, *p* = 0.0086) and in the dentate gyrus of the hippocampus (DG; *t*_14_ = 2.497, *p* = 0.0256) (Table 1).

**TABLE 1:** c-Fos densities (nuclei/mm^2^) counted within telencephalic brain regions of aged animals after intranasal administration of either vehicle (saline) or orexin-A (OxA) (50 μl, 100 μM). Among cortical brain regions, intranasal OxA significantly increased c-Fos expression within the agranular insular cortex and the prelimbic prefrontal cortex compared to intranasal saline administration. There was a trend (*p* = 0.0769) for decreased c-Fos expression in the infralimbic prefrontal cortex after intranasal OxA administration compared to intranasal vehicle. Intranasal OxA administration also significantly increased c-Fos expression within the claustrum and in the dentate gyrus of the hippocampus of aged animals compared to intranasal vehicle. Additional trends were observed in the retrosplenial cortex (*p* = 0.0988) and in the CA3 (0.0813) region of the hippocampus. *N* = 8/brain region/treatment condition. ***p < 0.001, **p < 0.01,*p < 0.05 versus saline.

**c-Fos density (nuclei/mm^2^)**		

**Brain region**	**Vehicle**	**100 μM OxA**
Piriform cortex	3770+407	4449422
Agranular insular cortex**	28551	699118
Prelimbic cortex***	2023120	3141232
Ventral orbital cortex	2754278	3574380
Infralimbic cortex (*p* = 0.0769)	2102111	1816100
Cla ustrum**	180596	2238104
Nucleus accumbens shell	62048	75466
Retrosplenial cortex (*p* = 0.0988)	1043102	1376158
Hippocampus-CA3 (*p* = 0.0813)	33+5	48+6
Hippocampus-CA1	5510	7314
Hippocampus-DG*	13812	18515

*p < 0.05; **p < 0.01; ***p < 0.001

### Effect of Intranasal OxA on c-Fos Expression in Parvalbumin (PV) + GABAergic Neurons of Aged Rats

**FIGURE 1:**
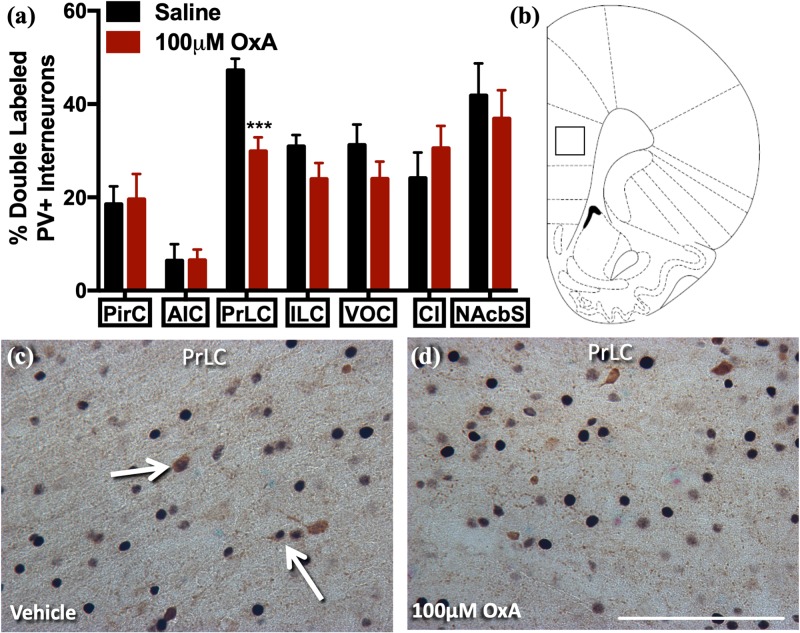
c-Fos expression in PV-positive GABAergic interneurons after intranasal OxA administration. Single-labeled and double-labeled PV neurons were counted within the same 0.7 mm × 0.7 mm reticle area as c-Fos so that direct comparisons could be established. **(A)** The percentage of PV + neurons coexpressing c-Fos relative to the total number of PV + neurons within the PirC, AIC, PrLC, ILC, VOC, Cl, NAcbS after treatment with intranasal saline (vehicle) or intranasal OxA (50 μl, 100 μM). Intranasal OxA significantly decreased c-Fos expression within PV-positive neurons in the PrLC. There were no other significant differences in any of the other cortical regions, claustrum, or nucleus accumbens shell. **(B)** Representative diagram indicating the approximate location (black-outlined square) within the PrLC where counts for c-Fos and PV + neurons and their photomicrographs were obtained. Bregma 2.70 mm **(C,D)** Representative photomicrographs highlighting typical dual-label immunohistochemistry for c-Fos nuclei (black) and PV neurons (brown) within the PrLC (arrows). PV, parvalbumin; OxA, orexin-A; PirC, piriform cortex; AIC, agranular insular cortex; PrLC, prelimbic cortex; ILC, infralimbic cortex; VOC, ventral orbital cortex; Cl, claustrum; NAcbS, nucleus accumbens shell. Scale bar represents approximately 100 μm (**D**). Error bars represent SEM. *N* = 8/brain region/treatment condition. ***p < 0.001 versus saline.

**FIGURE 2:**
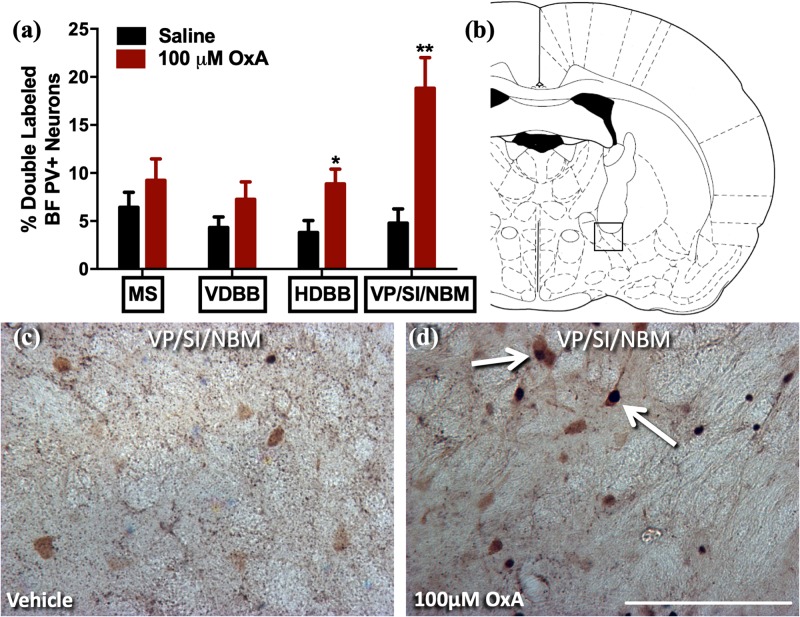
c-Fos expression in PV-positive GABAergic neurons of the basal forebrain after intranasal OxA administration. Single- and double-labeled PV neurons were counted within the same 0.35 mm × 0.35 mm area for each basal forebrain region so that direct comparisons could be established. **(A)** The percentage of PV + neurons coexpressing c-Fos relative to the total number of PV + neurons within the MS, VDBB, HDBB, and VP/SI/NBM after treatment with intranasal saline (vehicle) or intranasal OxA (50 μl, 100 μM). Treatment with intranasal OxA significantly increased c-Fos expression within PV-positive neurons of the HDBB and VP/SI/NBM. There were no other significant differences in any of the other basal forebrain regions. **(B)** Representative diagram indicating the approximate location (black-outlined square) within the VP/SI/NBM where counts for c-Fos and PV + neurons and their photomicrographs were obtained. Bregma –1.30 mm (**C**,**D**) Representative photomicrographs highlighting typical dual-label immunohistochemistry for c-Fos nuclei (black) and PV neurons (brown) within the VP/SI/NBM (arrows). PV, parvalbumin; OxA, orexin-A; BF, basal forebrain; MS, medial septum; VDBB, vertical limb of the diagonal band; HDBB, horizontal limb of the diagonal band; VP/SI/NBM, ventral pallidum/substantia innominata/nucleus basalis. Scale bar represents approximately 100 μm (**D**). Error bars represent SEM. *N* = 8/brain region/treatment condition. **p < 0.01, *p < 0.05 versus saline.

To analyze the effects of intranasal OxA on specific neuronal phenotypes in the cortex and basal forebrain, we stained cells for PV, a marker for fast-spiking GABAergic interneurons in the cortex (Hu et al., 2014). We found that intranasal OxA administration in aged animals significantly decreased c-Fos expression in PV + GABAergic interneurons of the prelimbic cortex (PrLC; *t*_14_ = 4.444, *p* = 0.0006) compared to treatment with intranasal saline. No significant effect was observed in any other cortical brain region (Figure 1). These observations suggest that intranasal OxA administration decreases activation of fast-spiking PV + GABAergic interneurons in the PFC. We also investigated the effects of intranasal OxA on PV + GABAergic neurons in different subregions of the basal forebrain of aged animals (Figure 2). Intranasal OxA selectively increased c-Fos expression within PV + GABAergic neurons of the horizontal limb of the diagonal band (HDBB; *t*_13_ = 2.580; *p* = 0.0229) and the contiguous ventral pallidum/substantia innominata/nucleus basalis region (VP/SI/NBM; *t*_13_ = 4.175, *p* = 0.0011). The total number of PV + neurons did not differ between OxA- and saline-treated animals in any brain region (Supplementary Table S2).

### Effect of Intranasal OxA on c-Fos Expression in ChAT + Neurons of Aged Rats

**FIGURE 3:**
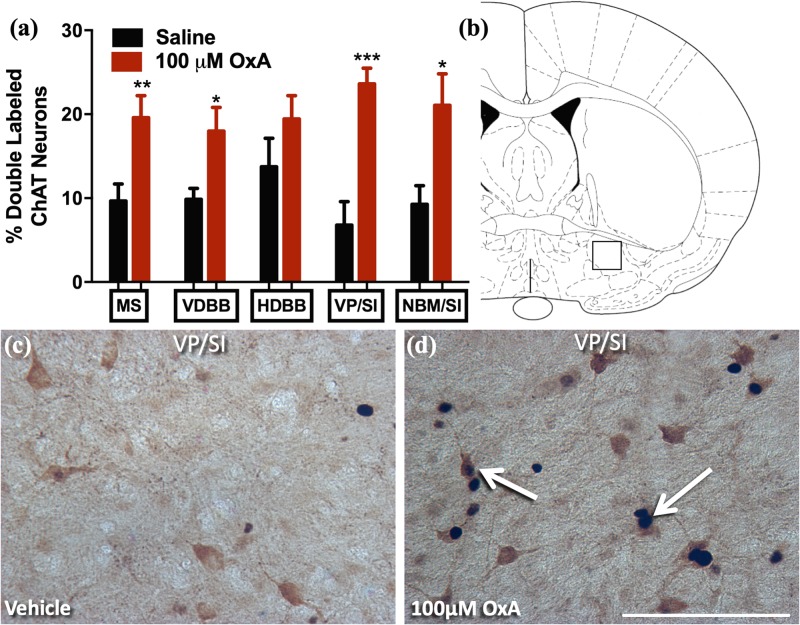
c-Fos expression in cholinergic neurons(ChAT +) of the basal forebrain after intranasal OxA administration. Single- and double-labeled ChAT neurons were counted within the same 0.45 mm × 0.45 mm area for each basal forebrain region so that direct comparisons could be established. **(A)** The percentage of ChAT + neurons coexpressing c-Fos relative to the total number of ChAT + neurons within the MS, VDBB, HDBB, VP/SI, and NBM/SI after treatment with intranasal saline (vehicle) or intranasal OxA (50 μl, 100 μM). Treatment with intranasal OxA significantly increased c-Fos expression within ChAT + neurons of the MS, VDBB, VP/SI, and NBM/SI. **(B)** Representative diagram indicating the approximate location (black-outlined square; Bregma –0.26 mm) within the VP/SI where counts for c-Fos and ChAT + neurons and their photomicrographs were obtained. **(C,D)** Representative photomicrograph highlighting typical dual-label immunohistochemistry for c-Fos nuclei (black) and PV neurons (brown) within the VP/SI (arrows). ChAT, choline acetyltransferase; OxA, orexin-A; MS, medial septum; VDBB, vertical limb of the diagonal band; HDBB, horizontal limb of the diagonal band; VP/SI, ventral pallidum/substantia innominata; NBM/SI, nucleus basalis/substantia innominata. Scale bar represents approximately 100 μm **(D)**. Error bars represent SEM. *N* = 8/brain region/treatment condition. ***p < 0.001, **p < 0.01, *p < 0.05 versus saline.

A large body of neurochemical and electrophysiological data indicates that locally administered OxA activates basal forebrain cholinergic neurons (Eggermann et al., 2001; Dong et al., 2006; Arrigoni et al., 2010; Fadel and Burk, 2010; Villano et al., 2017). Thus, we investigated if intranasal administration of OxA would elicit a similar response in aged animals. Statistical analysis revealed that intranasal OxA increased c-Fos expression in cholinergic (ChAT +) neurons of the medial septum (MS; *t*_14_ = 2.992, *p* = 0.0097), vertical limb of the diagonal band (VDBB; *t*_14_ = 2.613, *p* = 0.0204), ventral pallidum/substantia innominata (VP/SI; *t*_14_ = 4.980, *p* = 0.0002), and nucleus basalis/substantia innominata (NBM/SI; *t*_14_ = 2.702, *p* = 0.0172) of aged animals compared to treatment with intranasal saline (Figure 3). Overall, data are expressed as the percentage of ChAT + neurons that also express c-Fos (Figure 3A). The total number of ChAT + neurons did not differ between OxA- and saline-treated animals in any basal forebrain region (Supplementary Table S2).

### Intranasal OxA: Effects on PFC ACh and Glutamate Efflux

**FIGURE 4:**
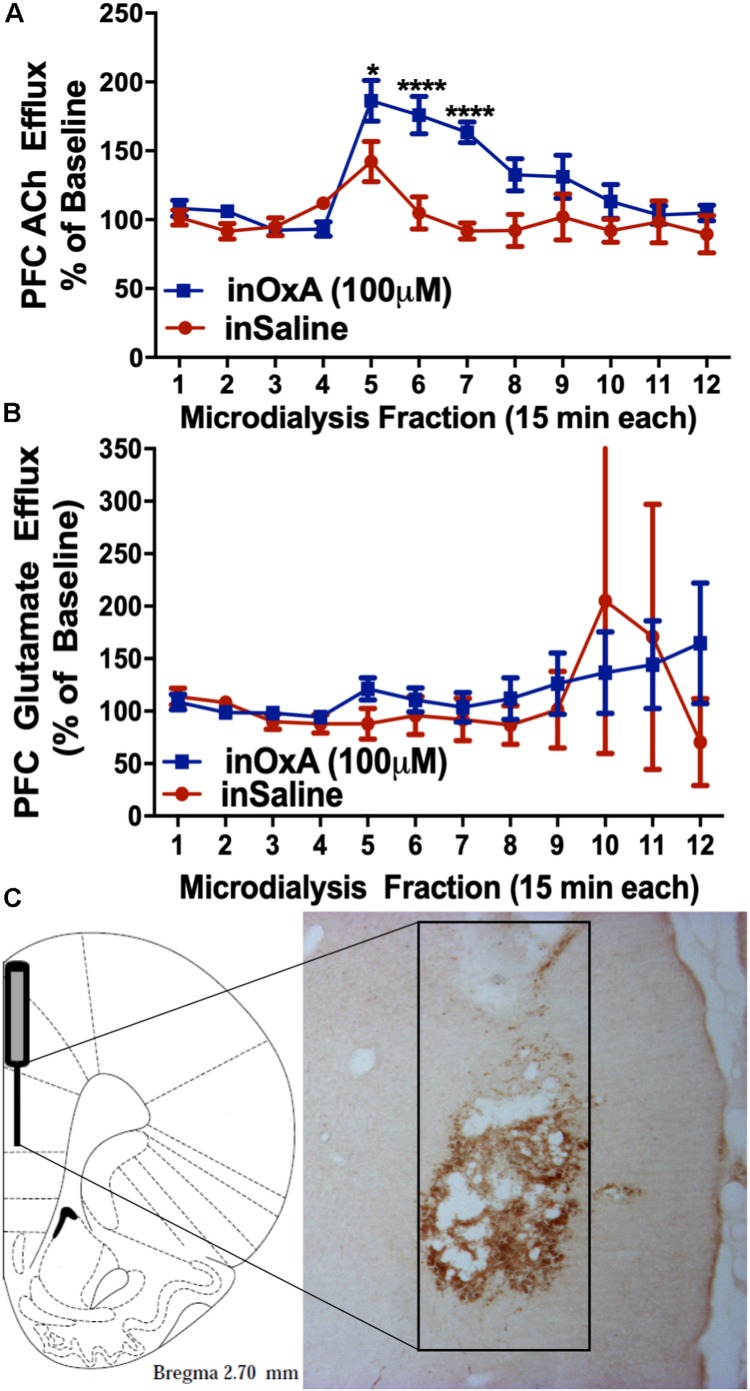
ACh and glutamate efflux in the PFC of aged animals after intranasal OxA administration. **(A)** Intranasal OxA (50 μl, 100 μM) treatment after baseline collections (arrow) significantly increased ACh efflux within the PFC compared to treatment with intranasal vehicle (saline). A significant increase in ACh efflux was observed from time points five through seven when compared with vehicle treatment. **(B)** Intranasal OxA treatment after baseline collections (arrow) did not significantly increase glutamate efflux within the PFC compared to treatment with intranasal saline. **(C)** Diagram indicating the approximate probe placement within the PFC for each of the animals that underwent microdialysis. Typical probe placement in the PFC is visualized through the presence of an AChE background stain. PFC, prefrontal cortex; ACh, acetylcholine; in, intranasal; OxA, orexin-A. Error bars represent SEM. *N* = 8/treatment condition. ****p < 0.0001, *p < 0.05 versus saline.

After observing increased c-Fos expression in the prelimbic PFC and in basal forebrain cholinergic neurons of aged rats, we then investigated the effects of intranasal OxA on PFC neurotransmission using microdialysis. We found that intranasal OxA significantly increased ACh efflux in the PFC, as indicated by a significant main effect of TIME (*F*_11_,_154_ = 10.53, *p* < 0.001), a significant main effect of TREATMENT (*F*_1_,_14_ = 10.22, *p* = 0.0065), and a significant TIME × TREATMENT interaction (*F*_11_,_154_ = 4.633, *p* < 0.0001). Further analysis with Holm-Sidak’s multiple comparison test showed that animals treated with intranasal OxA had significantly increased ACh efflux compared to animals treated with intranasal saline (Figure 4A) from collections five through seven (approximately 45 min). Intranasal OxA did not significantly alter PFC glutamate efflux in aged animals, as indicated by a two-way repeated-measures ANOVA that showed no significance for a TIME × TREATMENT interaction (*F*_11_,_154_ = 0.4881, *p* = 0.9085), a main effect of TIME (*F*_11_,_154_ = 0.6161, *p* = 0.9921) or a main effect of TREATMENT (*F*_1_,_14_ = 0.05874, *p* = 0.8120) (Figure 4B).

Histological analysis revealed that the 2-mm active membrane portion of the dialysis probe was centered in the prelimbic portion of the PFC with a small degree of overlap dorsally into the anterior cingulate cortex or ventrally into the infralimbic portion of the PFC (Figure 4C).

### Attentional Set-Shifting: Effects of Intranasal OxA on Attentional Function

**FIGURE 5:**
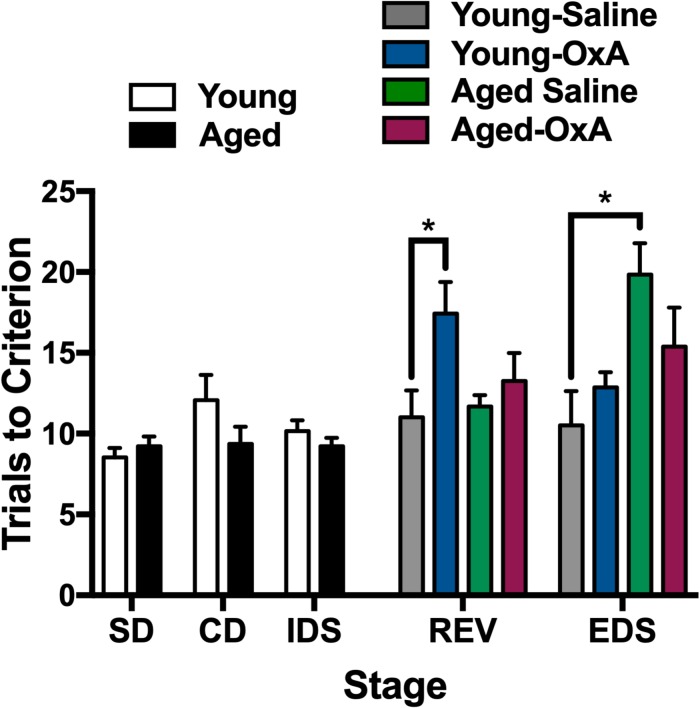
Intranasal OxA affects young and aged animals differently in an attentional-set shifting task. Animals were tested on the first three stages (SD, CD, IDS), then treated with intranasal saline or OxA, and subsequently tested on the final two stages (REV, EDS). Bars represent the average number of trials necessary to reach the criterion (six consecutive correct trials) for each stage of the task. The bars are the averages of 6–8 animals per group. Intranasal OxA significantly increased the total number of trials to criterion for young animals in the REV learning stage. Aged saline-treated animals are significantly impaired on the EDS stage compared to young saline-treated animals. Aged animals that received intranasal OxA did not perform significantly different from either young or aged rats that received saline. SD, simple discrimination; CD, compound discrimination; IDS, intradimensional shift; REV, reversal; EDS, extradimensional shift. Error bars represent SEM. *p < 0.05.

Attentional function appears to be modulated by the orexin system, in part, through actions on glutamatergic and cholinergic inputs that converge in the PFC (Fadel et al., 2005; Lambe et al., 2005; Huang et al., 2006). Prior work has previously demonstrated a capacity for OxA to increase cortical ACh levels and attentional processing, even among rats with lesions to the basal forebrain cholinergic system (BFCS) inputs to the PFC (Fadel and Burk, 2010; Zajo et al., 2016). These studies, combined with our studies highlighting increased PFC efflux of ACh after intranasal OxA administration, suggest that intranasal OxA may ameliorate age-related deficits in attention. To test the hypothesis that intranasal OxA improves age-related impairments in attentional performance, we used the attentional set-shifting paradigm along with intranasal administration of saline or OxA to determine the therapeutic potential of OxA (Figure 5). Two-tailed unpaired *t*-tests on the first three stages of the task (before treatment) indicated that there were no significant differences in performance between young and aged animals. A three-way ANOVA on the final two stages of the task (REV, EDS) revealed a significant stage × treatment interaction (*F*_1_,_48_ = 8.615, *p* = 0.0051), a significant stage × age interaction (*F*_1_,_48_ = 4.393, *p* = 0.0414), and a significant treatment × age interaction (*F*_1_,_48_ = 4.612, *p* = 0.0368). Holm-Sidak planned comparisons revealed that aged animals treated with saline were significantly impaired on the EDS stage of the task compared to young animals treated with saline (*p* = 0.0208). Intranasal OxA did not significantly improve performance in aged animals compared to aged animals treated with saline. However, performance in the EDS stage of aged animals treated with intranasal OxA was no longer significantly different from young saline- or young OxA-treated animals. Holm-Sidak multiple comparisons further revealed that young animals treated with OxA performed significantly worse on the REV stage compared to young animals treated with saline (*p* = 0.0409).

### Distribution of Intranasal 5(6)-FAM-(Glu^1^)-OxA in Aged Animals

**FIGURE 6:**
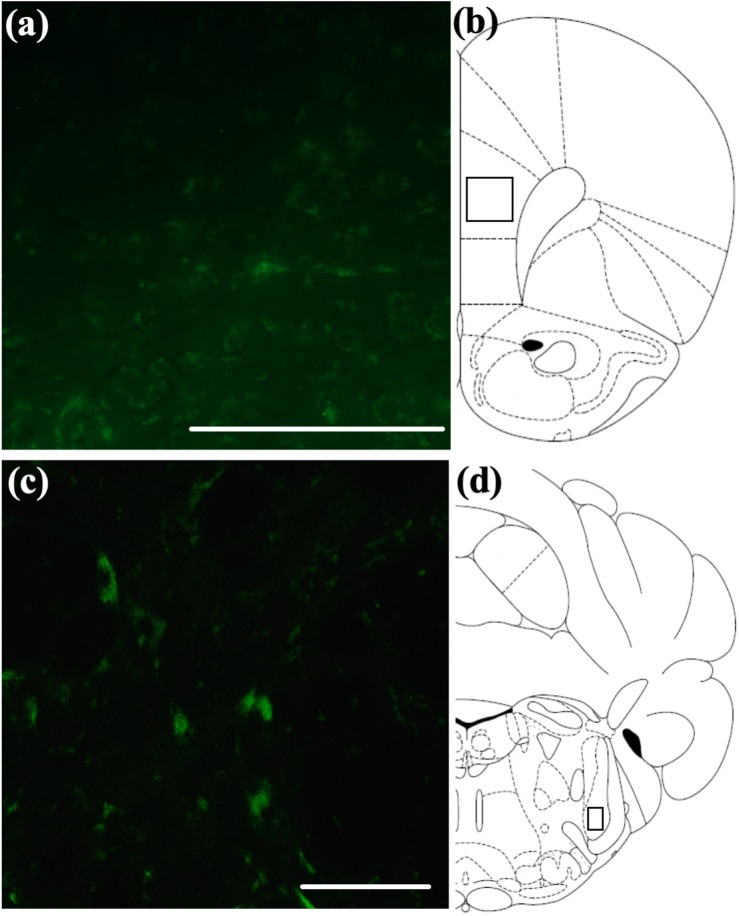
Qualitative visualization of a fluorescein-tagged OxA peptide in the brain after intranasal administration. **(A)** Representative localization of the fluorescein-tagged OxA peptide (50 μl, 500 μM) in the PrLC after intranasal delivery. **(B)** Representative diagram indicating the approximate region (black-outlined square) within the PrLC where fluorescence photomicrographs were obtained. **(C)** Representative localization of the fluorescein-tagged OxA peptide in the brain stem SpTrN after intranasal delivery. **(D)** Representative diagram indicating the approximate location (black-outlined square) within the SpTrN where fluorescence photomicrographs were obtained. Scale bars approximately 100 μm. OxA, orexin-A; PrLC, prelimbic cortex; SpTrN, spinal trigeminal nucleus.

Previous studies have suggested that intranasally administered peptides may access more caudal regions of the brain *via* extra-axonal diffusion along sensory trigeminal or olfactory pathways (Hanson and Frey, 2008; Chapman et al., 2013; Meredith et al., 2015; Spetter and Hallschmid, 2015). We have previously observed a distribution pattern of fluorescently labeled OxA in young animals consistent with this suggestion (Calva et al., 2018). To determine if a similar pattern is observed in aged animals and to provide descriptive background for the quantitative immunohistochemical, neurochemical, and behavioral results described above, we administered a fluorescein-tagged OxA peptide 5(6)-FAM-(Glu^1^)-OxA intranasally to aged rats. We observed fluorescent labeling in the principal sensory and spinal subdivisions of the trigeminal nucleus (Figures 6C,D), the sources of somatosensory innervation of the olfactory mucosa. The fluorescein-tagged OxA peptide was also detected in several telencephalic brain regions, including the medial PFC (Figures 6A,B). This overall pattern of distribution was similar to our previous observations in young animals. This distribution of fluorescence was not observed following intranasal vehicle (saline) administration (*n* = 4, aged; data not shown).

## Discussion

### Effects of Intranasal OxA on Neuronal Activation in the Cortex

Orexin neurons send extensive projections to cortical and subcortical brain regions that modulate attentional function. Consistent with the implications of this pattern of innervation, we observed that intranasal OxA elicited c-Fos expression in several brain regions and specific neuronal populations that play important roles in attention. For one, intranasal OxA significantly increased c-Fos expression in the agranular insular cortex. Importantly, this brain region is an important part of the circuit that controls interoceptive attention, a function crucial to an organism’s ability to detect changes to its internal physiological state. Prior work suggests that interoceptive awareness declines with age and may predict cognitive decline in the elderly (Khalsa et al., 2009; Murphy et al., 2018). In addition, while the basal forebrain provides diffuse cholinergic innervation of the neocortical mantle, reciprocal cortical modulation of the BFCS derives disproportionately from a few discrete cortical regions, which include the agranular insular cortex (Mesulam and Mufson, 1984; Zaborszky et al., 1997). Ultimately, the actions of intranasal OxA on the agranular insular cortex in aged animals suggest that OxA may enhance interoceptive attention during aging. In support of this, prior work from our lab has demonstrated that upregulation of orexin expression in aged animals restores behavioral deficits in feeding latencies when presented with a palatable food reward and enhances basal neurotransmission within the insular cortex (Hagar et al., 2017).

Orexin neurons also modulate exteroceptive attention, or attention to external cues, *via* extensive modulation of glutamatergic and cholinergic circuits that ultimately alter PFC activity (Fadel et al., 2005; Huang et al., 2006; Lambe et al., 2007; Zajo et al., 2016). Here, intranasal OxA administration significantly increased activation of the prelimbic PFC, a brain region known to play an important role in attentional shifting during a cognitively demanding task (Birrell and Brown, 2000; Dalley et al., 2001). Lastly, intranasal OxA administration also significantly increased c-Fos expression in the hippocampal dentate gyrus of aged animals. Previous work has demonstrated that selective blockade of the orexin-1 receptor in the dentate gyrus impairs long-term potentiation and memory acquisition and retrieval during a passive avoidance task, suggesting that orexin-1 receptors in the dentate gyrus may play an important role in learning and memory (Akbari et al., 2008, 2011).

### Effects of Intranasal OxA on Specific Neuronal Phenotypes in Aged Animals

Our studies here, in addition to our previous work in young animals, show that intranasal OxA administration decreases activation of fast-spiking PV + GABAergic interneurons in the PFC of aged rats (Calva et al., 2018). Mechanistically, decreases in c-Fos expression in prelimbic PV + interneurons after intranasal OxA administration may stem from increased activation of nucleus basalis/substantia innominata PV + GABAergic projection neurons, which tend to synapse directly onto PV + GABAergic interneurons in the cortex (Freund and Meskenaite, 1992; Henny and Jones, 2008). The functional implications of this apparent disinhibition of prelimbic PV + interneurons are unclear. PV + GABAergic interneurons in the PFC play important roles in promoting goal-driven attentional processing and cortical gamma power (Kim et al., 2015, 2016). Thus, diminished activation of these neurons might be expected to impair attentional function, although the dramatically different time scales underlying c-Fos expression and spike frequency make direct comparison of these measures difficult. Clearly, more work needs to be done to delineate the mechanisms underlying OxA effects on local PFC circuits and the implications for cognitive functions.

An extensive collection of prior work supports the role of orexin-cholinergic interactions in modulating attentional function. Here, we demonstrate that intranasal OxA administration in aged animals excites select subdivisions of the basal forebrain, namely, the MS, HDBB, and VP/NBM/SI subdivisions. These subdivisions of the basal forebrain cholinergic neurons send dense projections to the hippocampus, olfactory bulb, and neocortex/amygdala, respectively (Mesulam et al., 1983a, b). Importantly, cholinergic neurotransmission in these brain regions supports cognitive functions that decline during aging, specifically learning and memory, olfactory discrimination, and attention. Deficits in learning and memory are characteristically associated with aging. Previous work from our lab indicates that deficits involve intrinsic losses of orexin innervation to the medial septum (Stanley and Fadel, 2012). Ultimately, the ability of intranasal OxA to increase c-Fos expression in MS cholinergic neurons of aged animals suggests that intranasal OxA may increase cholinergic transmission to the hippocampus. A decline in the ability to discriminate between odors is becoming increasingly apparent in cognitive aging and may predict a decline from mild cognitive impairment (MCI) to full Alzheimer’s disease (Enwere, 2004; Kovács, 2004; Djordjevic et al., 2008; Sohrabi et al., 2012). The ability for intranasal OxA to improve olfactory dysfunction in neurological disorders with underlying deficits in orexin neurotransmission, namely, narcolepsy, indicates that intranasal OxA may be beneficial for treating age-related cognitive disorders (Baier et al., 2008). Finally, attentional deficits represent an important component of age-related cognitive decline. Lesions to the medial PFC and/or lesions to medial prefrontal cortical cholinergic projections produce deficits in attentional tasks and mimic the attentional deficits observed during aging (Barense et al., 2002; Zajo et al., 2016). Furthermore, administration of OxA directly into the basal forebrain ameliorates attentional deficits in animals with lesions to cholinergic projections to the medial PFC (Zajo et al., 2016). While we did not observe a significant effect of OxA relative to saline in our aged rats, OxA-treated old animals no longer performed significantly worse than young control rats.

### Intranasal OxA: Neuronal Activation and Neurotransmission in Aged Animals

The collective microdialysis results demonstrate that intranasal OxA administration rapidly and significantly increases ACh efflux within the PFC of aged animals, an area important for multiple aspects of cognitive function, especially attention. We have previously reported that intranasal OxA increases PFC ACh release in young rats (Calva et al., 2018), and while the magnitude of the effect seen here in aged animals was somewhat smaller, our data are consistent with the hypothesis that intranasal OxA can enhance PFC ACh efflux across the life span of these animals. Importantly, these large increases in PFC ACh efflux elicited by intranasal OxA administration are consistent with previous studies demonstrating that OxA increases cholinergic neurotransmission and that OxA administration enhances attentional processing (Fadel et al., 2005; Zajo et al., 2016). Indeed, high levels of ACh in the neocortex and hippocampus set the ideal dynamics for attentional processing and encoding of new information (Hasselmo and McGaughy, 2004). Additionally, previous work highlights that OxA is more potent than OxB at increasing the activity of basal forebrain cholinergic neurons and the subsequent release of ACh into the somatosensory cortex (Dong et al., 2006). Finally, our lab has demonstrated that basal forebrain ACh efflux elicited during feeding is eliminated when animals are pretreated with the orexin-1 receptor selective antagonist SB-334867 (Frederick-Duus et al., 2007). This evidence also indicates that orexin-mediated effects on cholinergic transmission in the cortex are primarily facilitated through the orexin-1 receptor. Our previous studies corroborate this idea as intranasal administration of the orexin-2 receptor agonist, [Ala^11^, D-Leu^15^]-OxB, selectively activates cholinergic neurons of the medial septum, an area which is not a major source of cholinergic input to the PFC (Calva and Fadel, 2018).

Qualitative visualization of (5(6)-FAM-(Glu^1^)-Hypocretin-1) in the PFC of aged animals would suggest that intranasal OxA may act directly on neurons within the PFC. However, unlike our observations in young animals (Calva et al., 2018), intranasal OxA did not significantly alter PFC glutamate efflux in aged animals. This may suggest that aged animals have a dysfunction in glutamatergic neurotransmission or glio-transmission as a large majority of the amino acid neurotransmitters sampled under basal conditions during *in vivo* microdialysis are derived from reverse transporter activity and/or from glial cells (Westerink, 1995; Timmerman and Westerink, 1997).

### Implications of Intranasal OxA for Age-Related Attentional Dysfunction

Intranasal OxA in young and aged animals elicited an interesting set of behavioral responses in the ASST. Aged Long-Evans rats have previously been shown to exhibit impaired performance on the EDS stage of the ASST experiment (Barense et al., 2002), a behavioral correlate of mPFC dysfunction (Birrell and Brown, 2000). Indeed, in our ASST paradigm, aged animals treated with intranasal saline were impaired on the EDS stage compared with young animals treated with saline. Altogether, this suggests that aged animals display impaired PFC-mediated attentional performance compared to young animals and that these deficits are extended across multiple strains (Barense et al., 2002). Furthermore, while intranasal OxA did not significantly improve performance in aged animals compared to aged saline-treated animals, EDS performance of the aged-OxA group was not significantly different from young saline- or young OxA-treated animals. Thus, intranasal OxA increases activation of basal forebrain cholinergic neurons and PFC ACh release in aged animals, and direct OxA application into the basal forebrain enhances cholinergic dependent attentional processing and ameliorates some attentional deficits in partial cholinergic-lesioned animals (Fadel et al., 2005; Zajo et al., 2016). Finally, intranasal OxA administration impaired the performance of young animals on the REV stage of the ASST task compared to young animals treated with intranasal saline. Ultimately, this suggests that acute administration of OxA to cognitively intact younger subjects does not enhance attentional performance and may overall significantly impair cognitive performance. Indeed, the large OxA-elicited increases in PFC ACh efflux that we observed in young animals may underlie this impaired performance, as cortical ACh levels are hypothesized to mediate attentional function in an “inverted U” dose-response curve (Bentley et al., 2011; Gritton et al., 2016). Thus, further increasing orexin levels in an intact animal may cause excess ACh release and thereby impair, rather than facilitate, attention. This notion is also supported by our observations that acute intranasal administration of OxA does not impair reversal learning in aged animals.

## Conclusion and Future Directions

In summary, these studies provide further evidence that intranasally administered OxA rapidly targets the brain and alters several anatomical, neurochemical, and behavioral correlates of cognitive functions, including in aged animals. In addition, OxA modulates performance in an attentional task that is consistent with effects on cholinergic transmission, suggesting that this peptide and route of administration may have therapeutic potential for treating cognitive disorders associated with diminished orexin levels. Ultimately, full dose-response studies of both acute and chronic intranasal OxA and other orexin receptor targets in the context of a broad range of cognitive and behavioral tasks will be required to elucidate the full therapeutic potential of intranasal OxA administration.

## Data Availability Statement

The datasets generated for this study are available on request to the corresponding author.

## Ethics Statement

The animal study was reviewed and approved by the University of South Carolina Institutional Animal Care and Use Committee.

## Author Contributions

CC performed surgery, animal training, histology, microdialysis, HPLC, statistical analysis, and wrote drafts of the manuscript. HF assisted with HPLC and immunohistochemistry. CC and JF collaborated on experimental design and interpretation. JF edited the final manuscript.

## Conflict of Interest

The authors declare that the research was conducted in the absence of any commercial or financial relationships that could be construed as a potential conflict of interest.

## Supplementary Material

FIGURE S1 Representative example of the testing arena used for the attentional set-shifting experiments. Animals were initially placed within the smaller third of the arena indicated by “Animal Start.” The removable divider was then removed, and the animals were allowed to discriminate between two separate bowls to ultimately dig for a palatable food reward.

TABLE S1 Task parameters for the attentional-set shifting paradigm. Tasks were performed in the following order: (1) simple discrimination, (2) compound discrimination, (3) Intradimensional shift, (4) reversal learning, and (5) extradimensional shift. Animals were counterbalanced such that half the animals started with odor as the relevant dimension and vice-versa. Positive cue pairs and their order were randomly determined ahead of time. Odor/media pairings remained the same for all animals tested. The following odor and media pairings were used: (1) Vanilla/Peppermint and GooglyEyes/Beads, (2) Strawberry/Cinnamon and Ribbon/Burlap, and (3) Banana/Hazelnut and Paper/Felt.

TABLE S2 Average numbers of ChAT + or PV + cell counts (± SEM) by brain region and treatment condition. None of the counts differed statistically as a function of treatment (vehicle vs. OxA; all *p*’s > 0.05).
