# Supplementary figures and images for: Effects of Intranasal Orexin-A (Hypocretin-1) Administration on Neuronal Activation, Neurochemistry, and Attention in Aged Rats
Source: Front Aging Neurosci. 2020 Jan 22;11:362. doi: 10.3389/fnagi.2019.00362 (PMC6987046; doi:10.3389/fnagi.2019.00362)

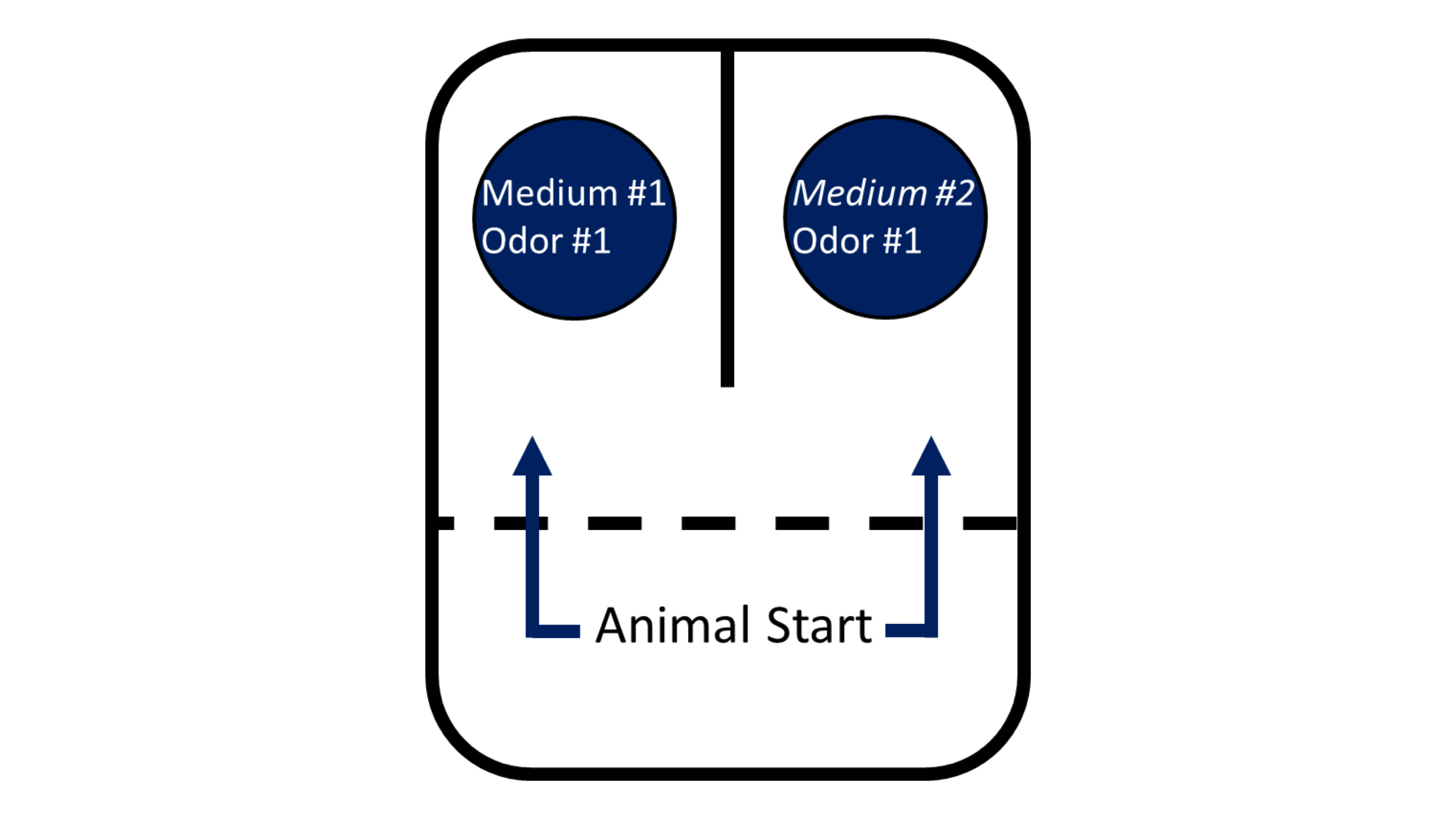

Supplement: FIGURE S1 — Representative example of the testing arena used for the attentional set-shifting experiments. Animals were initially placed within the smaller third of the arena indicated by “Animal Start.” The removable divider was then removed, and the animals were allowed to discriminate between two separate bowls to ultimately dig for a palatable food reward. [file Image_1.TIFF]

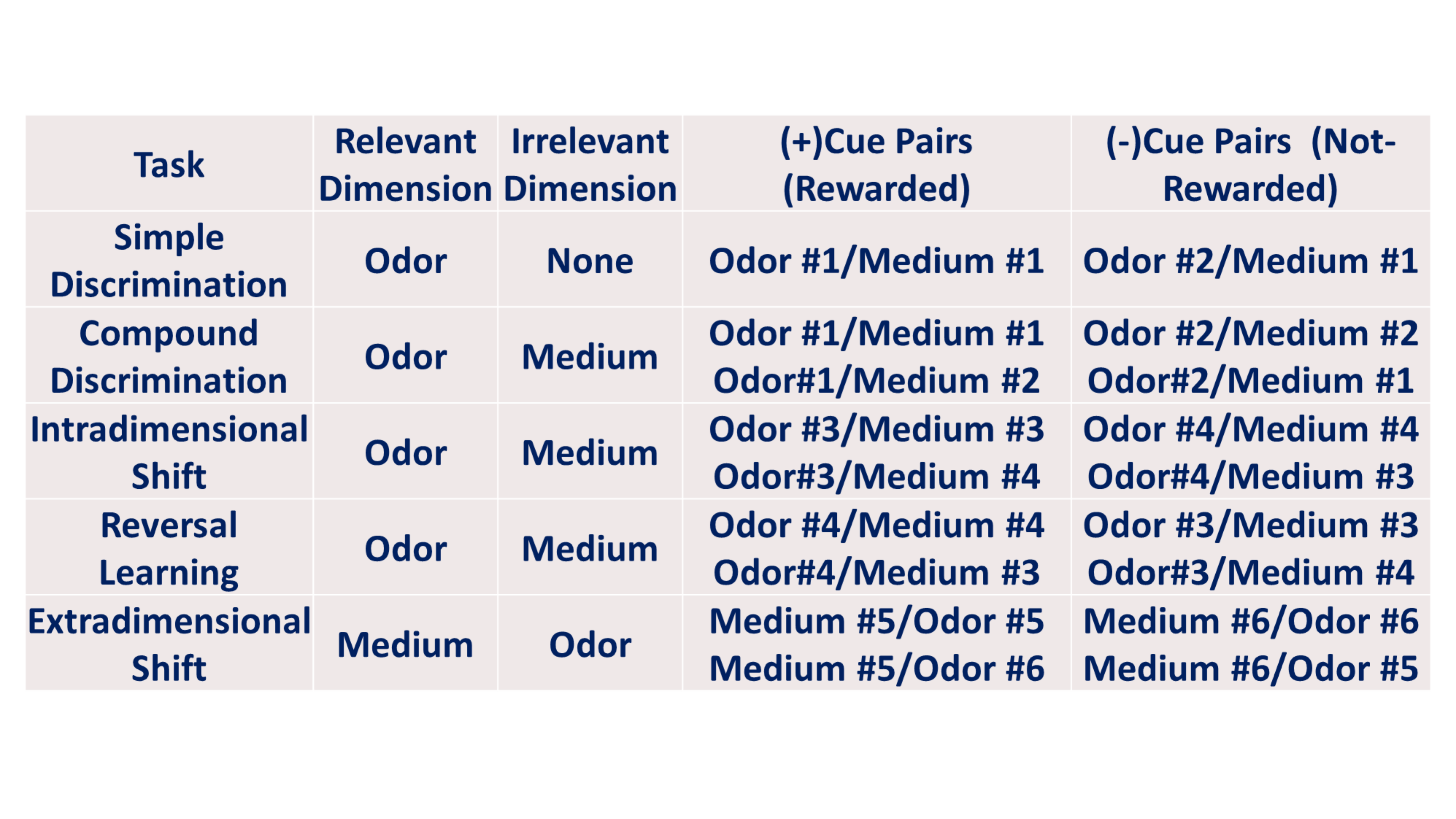

Supplement: TABLE S1 — Task parameters for the attentional-set shifting paradigm. Tasks were performed in the following order: (1) simple discrimination, (2) compound discrimination, (3) Intradimensional shift, (4) reversal learning, and (5) extradimensional shift. Animals were counterbalanced such that half the animals started with odor as the relevant dimension and vice-versa. Positive cue pairs and their order were randomly determined ahead of time. Odor/media pairings remained the same for all animals tested. The following odor and media pairings were used: (1) Vanilla/Peppermint and GooglyEyes/Beads, (2) Strawberry/Cinnamon and Ribbon/Burlap, and (3) Banana/Hazelnut and Paper/Felt. [file Image_2.TIFF]

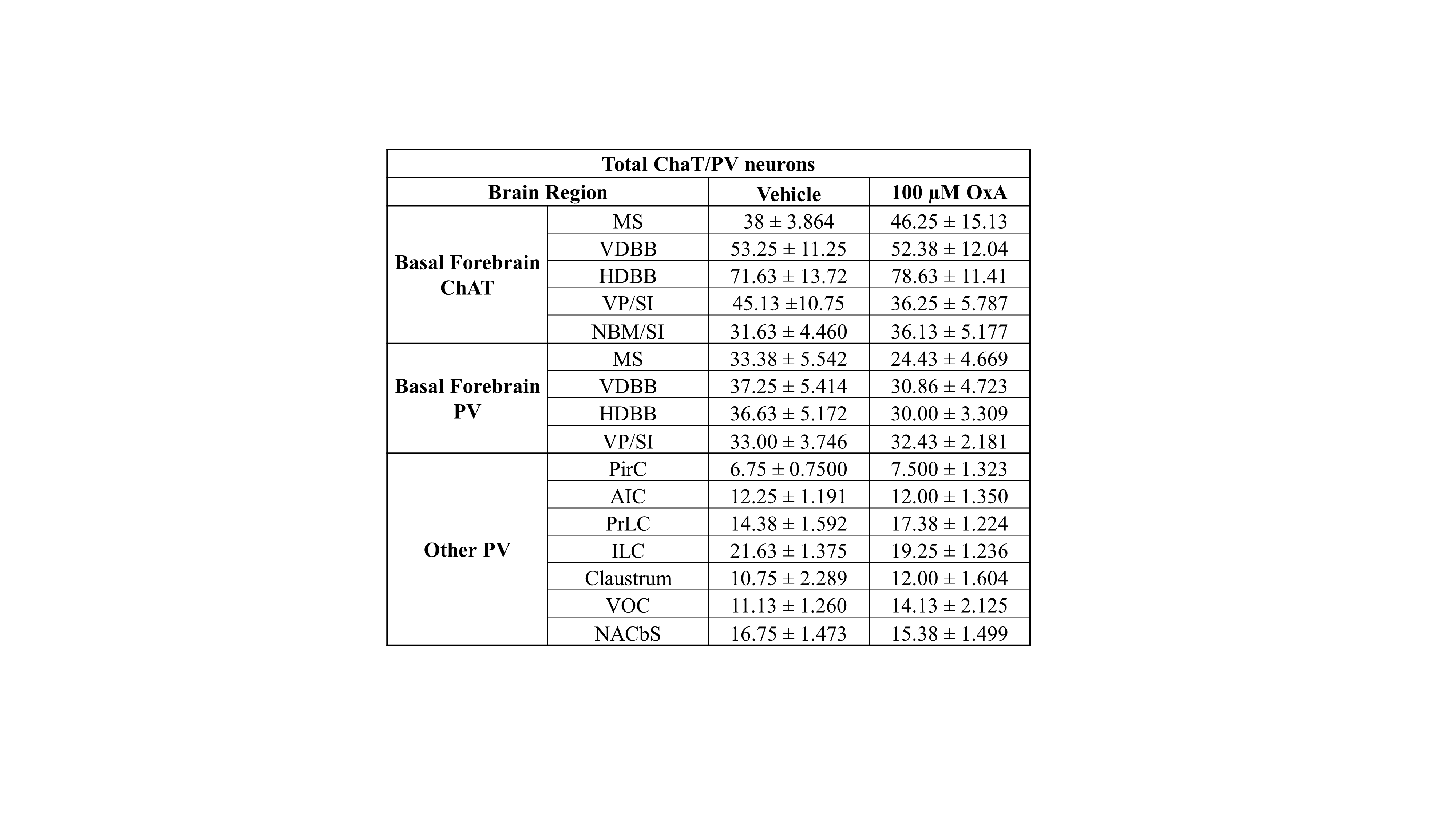

Supplement: TABLE S2 — Average numbers of ChAT + or PV + cell counts (± SEM) by brain region and treatment condition. None of the counts differed statistically as a function of treatment (vehicle vs. OxA; all p’s > 0.05). [file Image_3.TIF]
